# Policy priorities for strengthening smokeless tobacco control in Bangladesh: A mixed-methods analysis

**DOI:** 10.18332/tid/140826

**Published:** 2021-10-08

**Authors:** Rumana Huque, Zunayed Al Azdi, Aziz Sheikh, Jasjit S. Ahluwalia, Masuma P. Mishu, Ravi Mehrotra, Nasiruddin Ahmed, Linda Bauld, Syed Mahfuzul Huq, Syed Mahbubul Alam, Faraz Siddiqui, Sohel R. Choudhury, Kamran Siddiqi

**Affiliations:** 1 Department of Economics, University of Dhaka, Dhaka, Bangladesh; 2 ARK Foundation, Dhaka, Bangladesh; 3 Usher Institute, The University of Edinburgh, Edinburgh, United Kingdom; 4 Department of Behavioral and Social Sciences, Alpert Medical School, Brown University School of Public Health, Providence, United States; 5 Department of Health Sciences, Faculty of Sciences, University of York, Heslington, United Kingdom; 6 Centre for Health Economics, University of York, York, United Kingdom; 7 Department of Health Research, India Cancer Research Consortium, New Delhi, India; 8 8 Institute of Governance and Development, BRAC University, Dhaka, Bangladesh; 9 Country Office of World Health Organization, Dhaka, Bangladesh; 10 The Union, Dhaka, Bangladesh; 11 Department of Epidemiology and Research, National Heart Foundation Hospital and Research Institute, Dhaka, Bangladesh

**Keywords:** smokeless tobacco, tobacco control, regulation, tobacco tax, tobacco policy

## Abstract

**INTRODUCTION:**

Smokeless tobacco (ST) remains poorly regulated in Bangladesh. This study describes the prevalence and trends of ST use in Bangladesh, presents ST-related disease burden, identifies relevant policy gaps, and highlights key implications for future policy and practice for effective ST control in Bangladesh.

**METHODS:**

We analyzed secondary data from the two rounds (2009 and 2017) of The Global Adult Tobacco Survey, estimated ST-related disease burden, and conducted a review to assess differences in combustible tobacco and ST policies. In addition, we gathered views in a workshop with key stakeholders in the country on gaps in existing tobacco control policies for ST control in Bangladesh and identified policy priorities using an online survey.

**RESULTS:**

Smokeless tobacco use, constituting more than half of all tobacco use in Bangladesh, declined from 27.2% (25.9 million) in 2009 to 20.6% (22 million) in 2017. However, in 2017, at least 16947 lives and 403460 Disability-Adjusted Life Years (DALYs) were lost across Bangladesh due to ST use compared to 12511 deaths and 324020 DALYs lost in 2010. Policy priorities identified for ST control have included: introducing specific taxes and increasing the present *ad valorem* tax level, increasing the health development surcharge, designing and implementing a tax tracking and tracing system, standardizing ST packaging, integrating ST cessation within existing health systems, comprehensive media campaigns, and licensing of ST manufactures.

**CONCLUSIONS:**

Our analysis shows that compared to combustible tobacco, there remain gaps in implementing and compliance with ST control policies in Bangladesh. Thus, contrary to the decline in ST use and the usual time lag between tobacco exposure and the development of cancers, the ST-related disease burden is still on the rise in Bangladesh. Strengthening ST control at this stage can accelerate this decline and reduce ST related morbidity and mortality.

## INTRODUCTION

A diverse range of smokeless tobacco (ST) products are used worldwide. The pattern of ST consumption varies across countries due to differing socio-cultural norms, price, availability, accessibility, and tobacco control policies^1^. Betel quid with tobacco, zarda, gul, naswar, gutkha, and khaini are popular in South and South-East Asia. At the same time, the most commonly used ST products in other parts of the world include snus (Nordic countries and North America), chimó (Venezuela), nass (Kyrgyzstan, Uzbekistan), tambook (Chad, Sudan), and snuff (Ghana, Nigeria, South Africa)^2^. ST products are highly addictive, which results in high nicotine concentration and absorption. All ST products consumed in South and South-East Asia also contain carcinogenic substances like tobacco-specific N-nitrosamines (TSNAs). Many ST products are linked to wide-ranging harms, including specific types of cancers (head and neck), cardiovascular diseases, and poor perinatal outcomes^3^. ST use leads to an increased risk of cancers of the oral cavity, pharynx, and esophagus and increased deaths due to cardiovascular diseases^4,5^. Moreover, ST consumption during pregnancy is associated with low birth weight and stillbirths^6^.

The prevalence of ST use is high in Bangladesh. It ranks second in the world among high ST burden countries^7^, with 22 million (20.6%) adults consuming at least one form of ST product regularly^8^. Among adolescents aged 13–15 years, current ST use is 4.5%, with estimates of 5.9% and 2.0% in boys and girls, respectively^9^. Thus, ST use is higher than combustible tobacco use among adults8 and adolescents^9^ in Bangladesh.

ST products are popular, affordable, and readily available in Bangladesh, and their use is socially acceptable, also among women and young people^1,5^. However, low price, availability, and misconceptions regarding its beneficial health effects contribute to high ST use in Bangladesh^1,10^.

Bangladesh is a signatory to the World Health Organization (WHO) Framework Convention for Tobacco Control (FCTC). However, less attention has been paid to ST control compared to combustible tobacco^1^. With funding from the National Institute for Health Research (NIHR), UK, a group has been established by several academics, civil society organizations, and statutory bodies to address ST use in South Asia, including Bangladesh. The group is called ASTRA – Addressing Smokeless Tobacco and Building Research Capacity in South Asia^11^. On behalf of ASTRA, a mixed-methods study was conducted to describe ST prevalence and trend, estimate disease burden, and identify policy gaps, opportunities, and priorities to improve ST control in Bangladesh.

## METHODS

Our methodology involved five steps where we: 1) analyzed secondary data from two rounds of the Global Adult Tobacco Survey, 2009 and 2017 (GATS); 2) estimated the burden of disease due to ST consumption in adults and compared it with a 2010 estimate; 3) conducted a desk review of current tobacco control policies; 4) arranged an interactive workshop during the last quarter of 2019 to gather stakeholders’ views on gaps in the implementation of existing tobacco control policies for ST control; and 5) identified policy priorities using an online survey at the same period.

### Secondary data analysis

Data were analyzed from the GATS 2009 and 2017 to compare tobacco (total and prevalence) and ST consumption (total and prevalence) between the two rounds and compared ST use distribution with combustible tobacco.

### Estimating the disease burden

As part of estimating the global burden of ST use in 127 countries^12^, data were extracted for Bangladesh to calculate the disease burden due to ST use among adults. First, the country-specific prevalence of ST use in both men and women in Bangladesh was identified. Then, using the prevalence estimates and known disease risk (relative risks/odds ratios) estimates for oral, pharyngeal, and esophageal cancers and cardiovascular diseases^12^, the population attributable fraction (*PAF*) was estimated for ST use for Bangladesh. *PAF* would be the proportional reduction in disease or mortality if exposure were reduced to zero^13,14^. *PAF* was estimated for each disease for each country for both males and females, using the following formula:

*PAF=Pe (RRe–1)/[1 + Pe (RRe–1)]*

where *Pe*=prevalence of smokeless tobacco uses in that population and *RRe*=relative risk of disease (e.g. mouth cancer) due to SLT use.

Finally, these *PAFs* were used to estimate the disease burden attributable to ST for Bangladesh as a proportion of the deaths reported in the 2017 Global Burden of Disease study and Disability-Adjusted Life Years (DALYs) lost. Finally, the findings were compared with similar estimates using 2010 data reported by Siddiqi et al.^2^.

### Desk review

A desk review was carried out to assess differences in combustible tobacco and ST policies, if any. For this purpose, we reviewed tobacco-related policies, bills, acts, and legislative documents, and Statutory Regulatory Orders (SROs) issued in the last 15 years (2005–2019) by the Bangladesh Ministry of Health and Family Welfare (MOHFW) and Ministry of Finance. The documents had been collected from the National Tobacco Control Cell (NTCC) and Non-Communicable Disease Control Program (NCDC), MOHFW. In addition, two ministries’ websites (www.mof.gov.bd; www.mohfw.gov.bd) were also searched to extract relevant information.

### Priority setting workshop

We shared the findings of the above three methods of work at a stakeholders’ workshop. While the workshop’s primary aim was to share and validate the emerging results of desk review and secondary data analyses with the key stakeholders, the discussions during the workshop were expected to provide valuable data to identify the gaps in implementing tobacco control policies for ST control. Out of 44 invitees, a total of 38 policymakers, academics, advocates, and media representatives attended. The discussions were organized around five themes: fiscal policies, product regulation, education and awareness, cessation support, and knowledge gaps. We validated the policy gaps identified through the desk review with the experience and views of the participants in the workshop. Finally, we presented the data to and recorded comments of the participants, which were later triangulated with the findings from the desk review and the survey. A total of 44 key stakeholders were invited who are working in tobacco control in Bangladesh. The discussion was audio-recorded with the participants’ prior written consent, and two researchers took detailed notes. Consultations were analyzed thematically to identify key priorities for and challenges and opportunities in addressing ST use in Bangladesh.

### Online survey

Post-workshop, all 38 stakeholders were invited to complete an online survey to rank the policy options on a 5-point scale regarding their priority level. Stakeholder’s responses (n=36) were analyzed, and mean scores were estimated for the policy options and ranked accordingly.

## RESULTS

### Overall tobacco use

The two rounds of GATS indicated a decline in tobacco use among adults aged ≥15 years from 43.3% in 2009 to 35.3% in 2017, reflecting an 18.5% relative decline (Table 1). The absolute number of regular tobacco users also decreased over the period, from 41.3 million in 2009^15^ to 37.8 million in 2017^8^.

**Table 1:** Percentage of tobacco use by gender (GATS, 2009 and 2017)

*Tobacco use*		*2009*	*2017*	Percent change since 2009*
**Overall**	Overall tobacco use	43.3	35.3	-18.5
	Among males	58.0	46.0	-20.7
	Among females	28.7	25.2	-12.2
**Combustible** (cigarette)	Overall cigarette use	14.2	14.0	-1.4
	Among males	28.3	28.7	1.4
	Among females	0.2	0.2	0.0
**Combustible** (bidi)	Overall bidi use	11.2	4.8	-57.1
	Among males	21.4	9.7	-54.7
	Among females	1.1	0.6	-45.5
**Smokeless** (any)	Overall ST use	27.2	20.6	-24.3
	Among males	26.4	16.2	-38.6
	Among females	27.9	24.8	-11.1

* Percent change is estimated as (t_1_-t_2_)/t_1_, where t_1_ is the base value in year 2009 and t_2_ is the value in 2017.

### Combustible tobacco and ST use

ST use among adults (aged ≥15 years) declined from 27.2% in 2009 to 20.6% in 2017. In 2009, 25.9 million adults used ST, which represented 62.7% of all tobacco users, while in 2017, over 22 million adults used ST regularly, which constituted more than half (58.0%) of all tobacco users^16^. Among overall tobacco users, 18.7% were using betel quid with tobacco, and 3.6% were using gul in 2017^16^.

The extent of change in tobacco use over time varied by cigarette, bidi, ST products, and gender (Table 1). Overall combustible tobacco use declined from 23.0% in 2009 to 18.0% in 2017, while ST consumption over the same period decreased from 27.2% to 20.0%. ST use exceeded the prevalence of combustible tobacco use among adults in 2009^15^, and the pattern remained the same in 2017.

While bidi and ST use had declined, specifically among men, cigarette use has remained the same from 2009 to 2017^15^. Combustible tobacco use among men and ST use among women was still very high in 2017.

### Disease burden due to ST use

**Figure 1:**
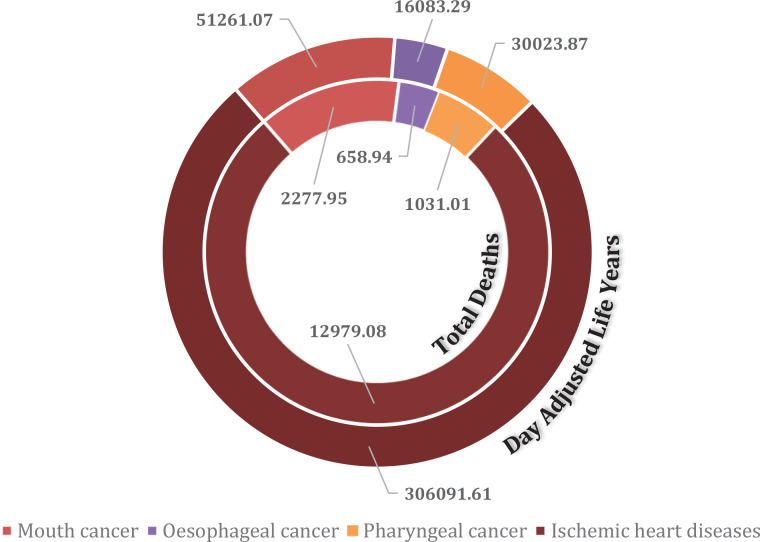
Burden of diseases due to ST use in Bangladesh (deaths per year, DALYs lost per year)

Contrary to the decline in ST use and in line with the usual time lag between tobacco exposure and the development of cancers, the ST-related disease burden is still on the rise. Using GATS 2009 data, Siddiqi et al.^2^ estimated that 12511 deaths and 324020 DALYs were lost in Bangladesh due to ST-related diseases. Data extracted from the estimation of the global disease burden^12^ suggests that in 2017, at least 16947 lives and 403460 DALYs were lost across Bangladesh due to oral, pharyngeal, esophageal cancers, and ischemic heart diseases that can be attributed to ST. Disease-specific DALYs and deaths that are attributed to ST use are shown in Figure 1.

### Tobacco control policies and gaps in ST control

**Figure 2:**
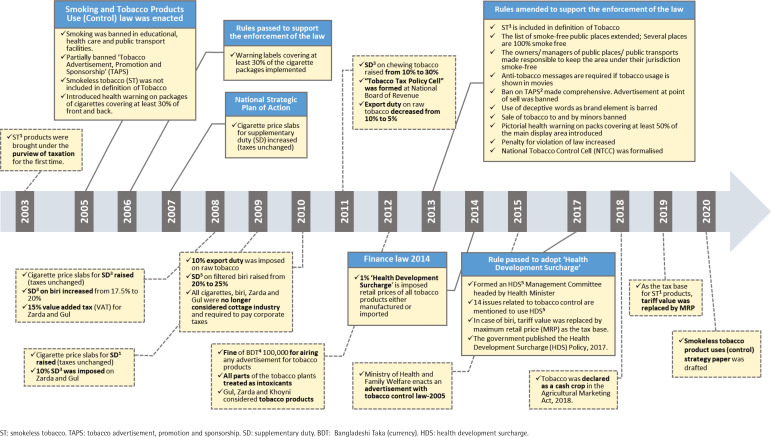
Tobacco control policies and law in Bangladesh

Smoking and Tobacco Product Usage Act in 2005 only covered combustible tobacco. The definition of ‘tobacco’ did not include ST as a tobacco product until 2013; the Act was not applicable for ST control (Figure 2). The Act was amended in 2013, and the Smoking and Tobacco Product Usage rules have been passed in 2015, which now covers both combustible tobacco and ST products^1^. A comparison of the tobacco control laws in Bangladesh for combustible tobacco and ST are summarized in Table 2. Almost all tobacco control laws apply to both combustible and ST products, except smoke-free laws. There is no law to prohibit the quantity of sale of any tobacco product. ST can be sold in any amount and is available in different-sized packs. This also restricts the law’s implementation of pack warning and banderols; warning labels and banderols cannot be added to small-sized ST packs, such as 5 g and 10 g packs. Tobacco packs – both combustible and ST – are not required to contain information on nicotine and tar content by law. Neither do the manufacturers are obliged to disclose the contents to the appropriate authorities.

**Table 2:** A comparative analysis of tobacco control laws in Bangladesh for both combustible tobacco and smokeless tobacco

*Sections under Smoking and Tobacco Product Usage Act 2005*	*Combustible*	*Smokelesstobacco*
Requirements to have nicotine and tar contents on the label produced locally	No	No
Requirements to have excise stamp, affixing banderols on the pack	Yes	Yes
Prohibition on quantity, i.e. sale less than 20 per pack	No	No
Smoke-free laws	Yes	Not applicable
Pictorial health warning on packs	Yes	Yes
Restriction on advertisement of tobacco	Yes	Yes
Restriction of promotion of samples	Yes	Yes
Prohibition of sale of tobacco to minors and by minors	Yes	Yes
Prohibition of storage, sale and distribution of tobacco in the immediate vicinity of educational institutions	No	No
Restriction on use in a public place and public transport	Yes	No

### Barriers and opportunities

Most of the participants in the stakeholders’ workshop acknowledged that despite the continuous government commitment for tobacco control, there remain gaps in implementation of and compliance to ST policies and regulations, requiring policymakers’ attention to reduce ST-related disease and economic burden in Bangladesh. Participants noted that ST was ingrained within Bangladeshi culture; hence awareness programs would likely only be effective if strictly enforced policies and regulations for ST control were in place. The large informal sector of ST manufacturing, storage, and distribution in Bangladesh makes it challenging to carry out product regulation through licensing and taxation. Other barriers to implementing policies against ST include a lack of governmental prioritization for ST control, the diverse nature of the ST, variations in the packaging, the complex supply chain with a limited understanding of the actors and their relationships, a lack of awareness of the ST-related health risks among policymakers and legislators, non-coordination among different ministries and departments, and an absence of evidence on cessation strategies. It was argued that obtaining information about the ingredients used in ST products and the date of manufacture is the people’s right, which may encourage users to quit ST use for a healthy life. It was also suggested that manufacturers notify the government about the levels of nitrosamines and tar they use in ST products. A multi-sectoral approach must coordinate decision-makers within different ministries, tobacco control advocates, and wider stakeholder groups.

### Policy priorities

The following priorities were identified (and ranked by the 36 workshop participants):

#### Fiscal policies

In all, 83% suggested imposing a specific tax on ST products as the most important fiscal measure to regulate ST products. A large proportion of participants (73%) also suggested increasing the current tax level for ST products, especially increasing the current health development surcharge on all tobacco products’ retail prices, either manufactured or imported (67%), as a priority. Designing and implementing a tax tracking and tracing system for ST (61%) should also be considered the government’s highest priority. Participants also suggested earmarking the tobacco taxes generated through the health development surcharge on tobacco products to provide alternative livelihoods to tobacco cultivators and laborers by ensuring the proper use of the budget from the 1% health development surcharge.

#### Cessation services

About two-thirds of the participants (61%) considered integrating treatment for tobacco dependence within the existing health systems as a very high priority. In addition, a national guideline for treating tobacco dependence, including smokeless tobacco (56%), comprehensive services to treat tobacco dependence (cessation clinics, quitlines) (53%), and incorporating treatment of tobacco dependence in health professionals’ curriculum (50%) were also considered highly important to reduce ST use.

#### Education and awareness

High priority for ST control was given to comprehensive media campaigns to raise public awareness (86%). Half of the participants suggested that anti-tobacco messages must be included within educational institutions and their curriculum. However, as a strong network of tobacco control advocates is already in place, only 44% of participants thought that an anti-tobacco advocacy coalition consisting of legislators, health professionals, journalists, cancer charities, and patient support groups was mandatory to control ST in the country.

#### Product regulation

Almost all agreed that formalizing the ST industry (e.g. licensing manufacturers, suppliers, and vendors) needed to be highly prioritized to control ST (89%). The formalization should be followed by the government formulating necessary policy to discourage the production and usage of ST and related crops (86%) and standardizing ST packaging and labelling, including pictorial warnings (81%). More than two-thirds of the participants pointed out that manufacturers must be obligated to list and disclose all ingredients to relevant authorities (70%).

#### Research priorities

The most important research priorities were identifying points in the ST supply chain where taxes can be levied (64%) and estimating the economic burden of ST in the country (53%). In addition, half of the participants thought it would be ideal to establish a technical group of experts to support the country’s national tobacco control cell. A few participants also emphasized the importance of assessing potential interventions to address ST use in different contexts.

## DISCUSSION

While ST use among adults declined between 2009 and 2017 in Bangladesh, there has been a considerable rise in ST-related morbidity and mortality over the same period. We found that Bangladesh has made notable progress in adopting ST control policies, including levying taxes on ST retail price, introducing pictorial health warnings, and banning advertising. However, implementation of and compliance with ST control policies are weak compared to regulatory efforts for combustible tobacco products. Moreover, most policies cannot be implemented adequately to address ST, mainly due to their diverse nature and manufacturing in informal settings. This implies that ST control policies and their implementation need to be strengthened. This priority setting exercise provided a list of policy actions to address ST use in Bangladesh, including introducing specific taxes and increasing the present *ad valorem* tax level for ST, especially increasing the health development surcharge, standardizing ST packaging, integration of ST cessation within existing health systems, comprehensive media campaigns, and an introduction of licensing of ST manufactures.

Taxation on ST remains low in Bangladesh compared to cigarettes. According to the budget speech presented in the Bangladesh National Parliament in 2020, in the fiscal year (FY) 2021, the supplementary duty (SD) of cigarettes for low-tier and three other tiers is 57% and 65% of maximum retail price (MRP), respectively, while the SD is 55% of MRP for zarda and gul. Imposing specific tax along with considerable increases in current *ad valorem* tax (supplementary duty) and price increase of all types of tobacco products to ensure that the affordability of heterogeneous ST products continues to decline was considered as a priority. Tax increases should be comparable across all tobacco products to reduce opportunities for substitution in response to changes in relative prices^17,18^. The government changed the tax base for zarda and gul from ex-factory price to MRP in FY 2020 and increased the price of a 10 g pack of zarda and of gul from 12 Taka (about US$0.14) and 6 Taka in FY 2019^19^ to 40 Taka and 20 Taka in FY 2021, respectively. However, this price increase will not impact ST consumption as many ST manufacturers are not registered and therefore not paying taxes and VAT to the government^1^. ST manufacturing units need to be formalized by bringing them under the licensing and registration process. This will also increase the tax revenue as ST taxation contributes only 0.22% of the tobacco tax revenue collected by the National Board of Revenue in Bangladesh^16^. Also, several non-manufactured ST products are available in the open market, which remain outside the tax net.

In addition to affixing banderols on ST products, designing and implementing an effective tax tracking and tracing system has been identified as a priority to monitor the real-time movement of the ST products through all stages of the supply chain and verify tax payments. Track and trace systems have been successfully used in Moldova, Turkey, and Brazil to increase tax collection, create new legal businesses, decrease tobacco use and improve public health^20,21^.

Pictorial health warnings need to be enforced for all types of products. This may require the standardization of ST packs to ensure compliance with the current law of 50% pack warning. Despite a high disease burden associated with ST use, cessation services are not available for ST users in routine healthcare at public facilities in Bangladesh1 and integrated policy including cessation support is warranted^22^. Also, as per the participants in the present study, a dearth of research on effective contextual intervention and lack of evidence on supply chain control remain to be addressed in the country, and matches the findings of a recent study^23^. The same study also suggests that the implementation of FCTC has not been in alignment with the requirements of ST control policies and vice versa.

The two rounds of GATS highlighted both a sharp decline in bidi use and a considerable reduction in ST use in Bangladesh over the last ten years. However, cigarettes, which remain a more expensive form of tobacco, remained the same over the years. This indicates that sustained growth of the market of cheap cigarettes and inappropriate tobacco tax structure combined with socioeconomic development might have resulted in brand and product switching rather than reducing or quitting tobacco use when taxes and prices increase^24,25^.

Previous studies also suggested that the ST-related disease burden is substantial, with at least 2.5 million DALYs lost in 2017^12^. However, efforts to control ST use, in general, remain suboptimal compared to cigarette consumption^26^. The policy priorities identified in our analysis are consistent with global priorities for ST control^3,27^. Further research is needed to assess the impact of these policies and interventions on health. We recommend a multi-sectoral approach and a comprehensive media campaign to address public health issues like ST.

### Limitations

Our study has several limitations. First, a selected number of participants were invited for the workshop and the online survey, who had been purposively chosen from the wider stakeholder group (health advocates, healthcare providers, media representatives, and researchers) working on tobacco control. However, the stakeholders had long working experience in the field, and many of them had closely worked with the policymakers in developing tobacco control policies in Bangladesh. Second, the GATS 2017 full data set was not available during the study period, which restricted further analysis of the data. Despite the limitations, this paper is the first attempt to consolidate and coordinate research and advocacy expertise in ST use in Bangladesh and identified policy priorities for ST control in Bangladesh.

## CONCLUSIONS

Smokeless tobacco consumption has declined in Bangladesh since 2009, and strengthening ST control at this stage can accelerate this decline and reduce ST-related morbidity and mortality, including oral cancer deaths, over the coming decades. Strict enforcement of the law, stringent tax measures, and cessation support need to be provided by strengthening the tobacco control program.

## Data Availability

The data supporting this research are available from the authors on reasonable request.
